# All-diamond optical assemblies for a beam-multiplexing X-ray monochromator at the Linac Coherent Light Source

**DOI:** 10.1107/S1600576714013028

**Published:** 2014-08-01

**Authors:** S. Stoupin, S. A. Terentyev, V. D. Blank, Yu. V. Shvyd’ko, K. Goetze, L. Assoufid, S. N. Polyakov, M. S. Kuznetsov, N. V. Kornilov, J. Katsoudas, R. Alonso-Mori, M. Chollet, Y. Feng, J. M. Glownia, H. Lemke, A. Robert, M. Sikorski, S. Song, D. Zhu

**Affiliations:** a Advanced Photon Source, Argonne National Laboratory, Argonne, Illinois, USA; b Technological Institute for Superhard and Novel Carbon Materials, Troitsk, Russian Federation; c Skobeltsyn Institute of Nuclear Physics, Lomonosov Moscow State University, Moscow, Russian Federation; d Illinois Institute of Technology, Chicago, Illinois, USA; e Linac Coherent Light Source, SLAC National Accelerator Laboratory, Menlo Park, California, USA

**Keywords:** diamond, X-ray monochromators, beam multiplexing

## Abstract

A double-crystal diamond (111) monochromator recently implemented at the Linac Coherent Light Source (LCLS) enables splitting of the primary X-ray beam into a pink (transmitted) and a monochromatic (reflected) branch. The first monochromator crystal, with a thickness of ∼100 µm, provides sufficient X-ray transmittance to enable simultaneous operation of two beamlines. This article reports the design, fabrication and X-ray characterization of the first and second (300 µm-thick) crystals utilized in the monochromator and the optical assemblies holding these crystals. Each crystal plate has a region of about 5 × 2 mm with low defect concentration, sufficient for use in X-ray optics at the LCLS. The optical assemblies holding the crystals were designed to provide mounting on a rigid substrate and to minimize mounting-induced crystal strain. The induced strain was evaluated using double-crystal X-ray topography and was found to be small over the 5 × 2 mm working regions of the crystals.

## Abstract 2

All-diamond optical assemblies holding state-of-the-art type IIa diamond crystals enable the construction of a beam-multiplexing X-ray double-crystal monochromator for hard X-ray free-electron lasers. Details on the design, fabrication and X-ray diffraction characterization of the assemblies are reported.

## 1. Introduction   

Top-quality type IIa diamond crystals suitable for advanced applications in X-ray crystal optics at synchrotrons and X-ray free-electron lasers (XFELs) have recently become available as a result of refinements in the high-pressure high-temperature (HPHT) crystal synthesis method (Burns *et al.*, 2009 ▶; Polyakov *et al.*, 2011 ▶; Sumiya & Tamasaku, 2012 ▶). Diamond crystal plates with (111) surface orientation are of primary importance for front-end diffracting X-ray optics owing to a greater intrinsic energy bandwidth of the 111 Bragg reflection and the resulting flux of the reflected X-rays compared with those of higher-order reflections. However, production of (111) crystal plates with large defect-free regions is a more challenging task than production of plates of other orientations close to the [001] direction. This is because (i) the (001) diamond HPHT growth sector has the best crystal quality and the lowest impurity concentration; and (ii) the {111} crystal faces are the most resistant to polishing since these planes have the highest atomic density.

**Figure 1:**
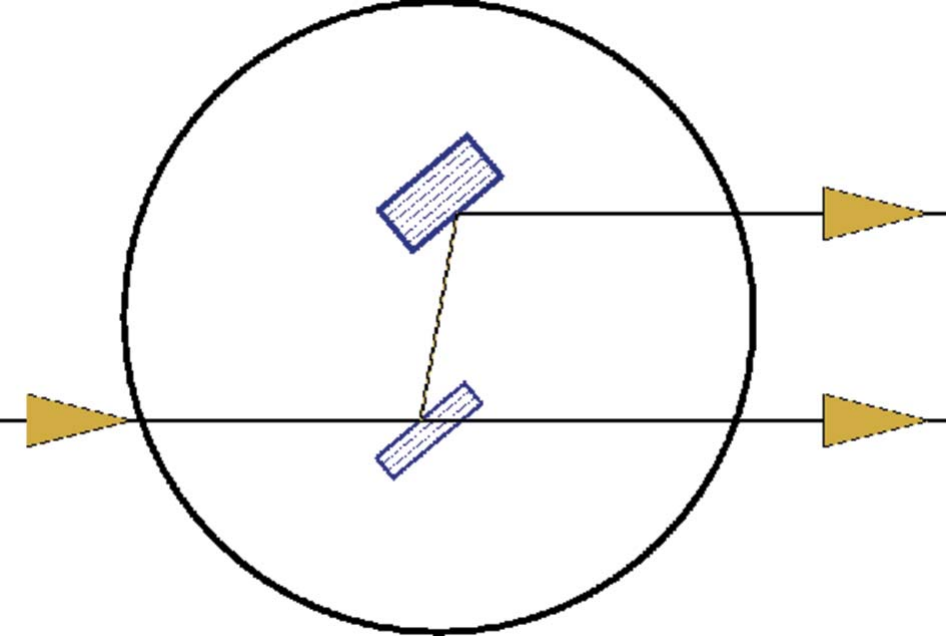
Scheme of a double-crystal beam-multiplexing monochromator in Bragg reflection geometry. The first crystal is sufficiently thin to avoid substantial losses in the transmitted branch due to photoabsorption.

The primary application of diamond (111) crystal plates in X-ray optics is as high-heat-load monochromators at synchrotron undulator beamlines (*e.g.* Grübel *et al.*, 1996 ▶; Fernandez *et al.*, 1997 ▶; Yabashi *et al.*, 2007 ▶). A scheme of a double-crystal monochromator in Bragg reflection geometry is shown in Fig. 1 ▶. If the first crystal is made sufficiently thin, the beam transmitted through it can be used in a parallel experiment downstream, *i.e.* beam multiplexing. Utilization of X-ray monochromators with low-absorption crystals for beam multiplexing was pioneered at the TROIKA beamline at the ESRF (European Synchrotron Radiation Facility, Grenoble, France)  (Als-Nielsen *et al.*, 1994 ▶; Grübel *et al.*, 1994 ▶, 1996 ▶). This beam-multiplexing approach gains even more importance for hard X-ray free-electron lasers, which recently redefined the frontiers of X-ray sciences (Emma *et al.*, 2010 ▶; Ishikawa *et al.*, 2012 ▶; Amann *et al.*, 2012 ▶). Using a single straight electron trajectory of an XFEL, multi-user operations cannot be achieved as is commonly accomplished at storage-ring-based synchrotron sources. However, multiplexing can be performed by means of X-ray optics, which enables simultaneous delivery of portions of the XFEL beam to several experiments. This yields an increase in the total number of performed experiments and thus reduces the high XFEL operating cost per experiment.

Highly developed crystal fabrication and processing methods for silicon have led to a few attempts to use Si crystals for beam-multiplexing XFEL monochromators. Greater X-ray absorption in Si requires utilization of ultra-thin crystals (thicknesses ∼5–10 µm) to achieve sufficient transmittance of the XFEL beam (30–80%) over a photon energy of 4–10 keV. High-quality ultra-thin Si crystals for XFEL beam-multiplexing monochromators can be manufactured using state-of-the-art processing methods (Feng *et al.*, 2012 ▶; Osaka *et al.*, 2013 ▶); however, such ultra-thin crystals have been found to be unstable in the Linac Coherent Light Source (LCLS) beam  (Feng *et al.*, 2012 ▶; Feng, Zhu *et al.*, 2013 ▶). It was found that XFEL pulse-induced distortions were very long lived, with a time constant greater than 100 ms, thus making these crystals unsuitable for operation at the repetition rate of the LCLS (120 Hz). The angular distortions were estimated to be greater than or comparable to the Darwin width of the Si 111 reflection.

Similar levels of transmittance can be achieved with crystal thicknesses of 50–100 µm in the case of diamond owing to its lower X-ray absorption. The greater crystal thickness and Young’s modulus for diamond ensure a substantially greater stiffness of the crystal. Diamond crystals with such thicknesses are expected to be more stable in the XFEL beam if an appropriate crystal mounting scheme is provided.

Because of the small divergence (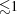
 µrad r.m.s.) of the XFEL beam, its wavefront is particularly sensitive to imperfections of X-ray optics. Disturbances of the wavefront introduced by the optics should be much less than or at least comparable to the beam divergence, which is a challenging requirement, especially for diamond crystal optics prone to crystal defects. Along with the presence of intrinsic defects, a mounting-induced crystal strain is another main factor that leads to deterioration of the crystal diffraction performance, which disturbs the radiation wavefront. The mounting solution previously developed by some of the co-authors for the diamond (001) self-seeding XFEL monochromator (Stoupin *et al.*, 2013 ▶; Shu *et al.*, 2013 ▶) was found to show limitations in heat transfer between the crystal plate and the graphite holder where the plate was loosely mounted (Feng, Alonso-Mori *et al.*, 2013 ▶).

In this work we report on the production of 100 µm-thick and 300 µm-thick type IIa HPHT diamond crystal plates with (111) orientation. Each crystal plate has a region with a low concentration of defects (working region) of about 5 × 2 mm, sufficient for use in XFEL optics. More importantly, we present a solution for strain minimization as a special mounting of such crystals on a rigid substrate. All parts of the optical assembly holding each crystal plate were fabricated out of diamond, which improved the heat transfer and radiation hardness of the device. The mounting-induced strain was evaluated using double-crystal X-ray topography in rocking curve diffraction imaging mode. The mounting-induced crystal distortion (1.5 µrad r.m.s. variation in the rocking curve peak position across the working region) was substantially smaller than the full width at half-maximum (FWHM) of the rocking curve width (25–30 µrad) and still comparable to the divergence of the XFEL beam. The two assemblies were installed in the double-crystal monochromator at the XPP instrument of the LCLS and successfully tested (Zhu *et al.*, 2014 ▶). The monochromator was operated in Bragg reflection geometry, as shown in Fig. 1 ▶. The capability of splitting the XFEL beam into a pink (transmitted) and a monochromatic (reflected) branch was demonstrated, which enables the use of the XFEL beam in two experiments simultaneously.

## 2. Diamond (111) crystal plates   

Type IIa diamond single crystals were grown at the Technological Institute for Superhard and Novel Carbon Materials (TISNCM) using the temperature-gradient method at high static pressure and high temperature (*e.g.* Blank *et al.*, 2007 ▶; Polyakov *et al.*, 2011 ▶). The temperature of crystallization was 1733 K at a pressure of 5.5 GPa. After the crystallization process, the diamond crystals were cut by a laser along the 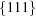 crystal plane with a ∼2° angular offset from the plane. The angular offset was introduced to facilitate mechanical polishing of the crystal plates. The plates were polished to a micro-roughness of ∼10 nm (r.m.s.).

Preliminary selection of crystal plates with a low density of crystal defects was made using Lang X-ray topography.

For the final selection of diamond crystal plates, white-beam X-ray topography was performed at the MRCAT 10BM (bending magnet) beamline  (Kropf *et al.*, 2010 ▶) of the Advanced Photon Source. White-beam X-ray topographs were obtained in Laue (transmission) geometry on the diamond plates enclosed in Kapton film holders. The source-to-sample distance was 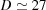
 m, the effective source size at the beamline was 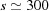
 µm, and the distance from the sample to the observation plane was 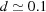
 m. Thus, the expected spatial resolution in the observation plane was 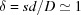
 µm, which was comparable to the photographic resolution of the utilized X-ray film (AGFA STRUCTURIX D3-SC).

**Figure 2:**
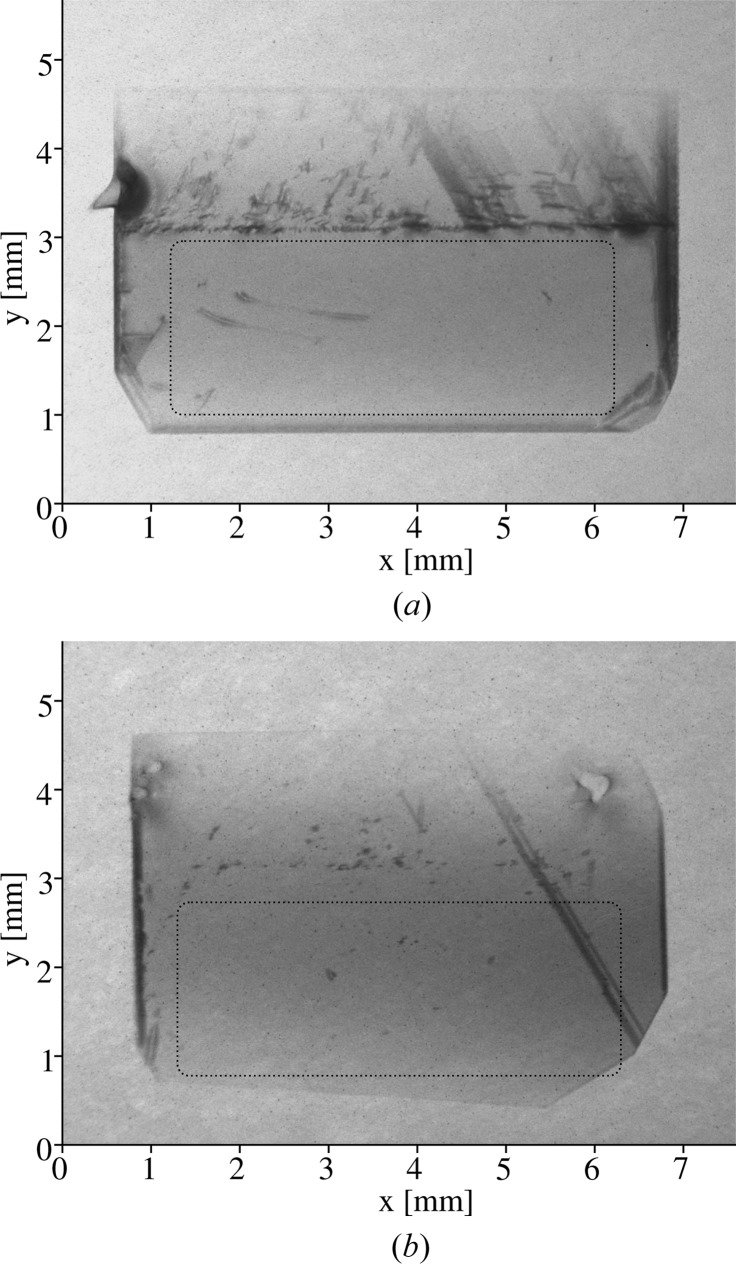
White-beam X-ray topographs of the selected diamond (111) crystal plates: (*a*) 300 µm-thick plate (second monochromator crystal) and (*b*) 100 µm-thick plate (first monochromator crystal). The images are obtained from the 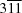 diamond reflection in Laue geometry. The dotted boxes illustrate the 5 × 2 mm working regions, containing only a few defects.

We note that the image resolution was somewhat reduced as a result of the crystal instability caused by heat load of the X-rays during the shortest available exposure time (∼2 s). Multiple images were taken to mitigate this problem. High-quality topographs were obtained from the 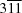 reflections. They are shown in Figs. 2 ▶(*a*) and 2 ▶(*b*) for the selected 300 µm-thick and 100 µm-thick crystal plates, respectively.

The dotted boxes in Figs. 2 ▶(*a*) and 2 ▶(*b*) show the 5 × 2 mm working regions, originated for the most part from the (001) growth sector, with a low concentration of defects. A few defects of about 10 µm in size are noticeable in the working regions of the crystals. These can be attributed to minor dislocations and micro-inclusions. The diagonal lines represent stacking faults that are likely to originate at the growth sector boundaries. Nevertheless, the crystals are of remarkable quality in the 5 × 2 mm region.

## 3. Crystal mounting method   

The relative intrinsic energy bandwidth of X-ray Bragg reflection is a very small quantity (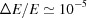
–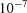), which is related to the small intrinsic angular width 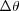 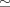 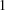
–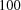
 µrad (except for Bragg backscattering cases where 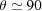
°) as
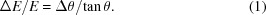



Since the relative energy bandwidth is small, a similarly small crystal strain 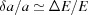 (*a* is the crystal lattice parameter) can distort the shape of the intrinsic curves and reduce the performance of the crystal as a monochromator. Thin Bragg crystals are particularly affected since relative deformations of the crystal are larger for a given applied stress (force). The case in which a thin crystal has a fixed contact point with a holder (achieved *via* bonding using glue, tape or even grease) can be distinguished in Bragg diffraction from the situation where the crystal has a nearly free boundary (*i.e.* motion of the crystal is constrained only by friction against a crystal holder). Such a nearly free boundary condition is appropriate for certain applications; however, in this case the crystal is very susceptible to vibrations. Also, heat transfer between the crystal and the holder could be limited because of the reduced surface contact. These factors may severely deteriorate the diffraction performance of the crystal under an intense incident X-ray beam.

**Figure 3:**
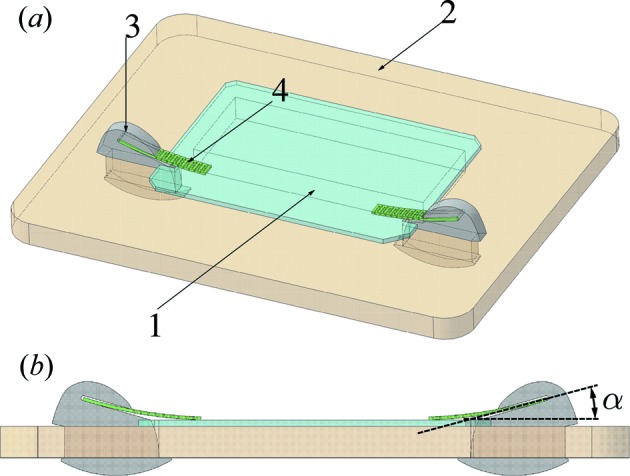
Scheme of the all-diamond optical assembly. (*a*) Perspective view showing the components of the assembly: a type IIa HPHT diamond (111) crystal plate 1, a CVD diamond substrate 2, diamond restrainers 3, and perforated CVD diamond springs 4. (*b*) Side view showing the orientation of the grooves in the restrainers for insertion of the CVD diamond spring. The groove is at an angle α relative to the surface of the optical element.

In this work, an alternative approach to mounting a diffracting crystal is explored, which involves a gentle pressure (a spring force) applied to the crystal placed on a rigid substrate. This force is applied in a controlled manner, such that the resulting strain is evaluated and limited to desired specifications. The optical assembly is shown schematically in Fig. 3 ▶. In order to minimize issues related to differential thermal expansion, and to improve radiation hardness and heat transfer, all parts of the assembly were manufactured out of diamond materials. To improve heat transfer between the substrate and the crystal their surfaces in contact were polished.

A high-quality type IIa HPHT diamond (111) crystal plate was mounted on a substrate fabricated out of polycrystalline chemical vapor deposited (CVD) diamond. The substrate is sufficiently thick (500 µm) to provide rigid support for the diamond plate. The surface of the substrate in contact with the diamond plate was polished to ∼10 nm (r.m.s.) micro-roughness. The substrate has two small rectangular openings for insertion of restrainers made of low-quality type IIa HPHT diamond and one large rectangular window (5 × 2 mm) for passage of X-rays transmitted through the diamond plate. The diamond plate has two small rectangular cuts on the sides such that the restrainers prevent lateral displacement of the plate on the substrate. The restrainers have grooves for insertion of 15 µm-thick CVD diamond stripes (CVD springs) that provide a gentle force on the diamond plate, thus preventing its vertical displacement. The force acting on the diamond is
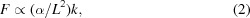
where α is the bending angle of the CVD spring as shown in Fig. 3 ▶(*b*), *L* is the length of the spring and *k* is the flexural stiffness of the spring. The spring can be approximated by a cantilever beam with an area moment of inertia *I*, such that 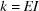, where *E* is Young’s modulus. Thus, for a fixed angle α (in our design 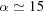
°) the force can be controlled by either variation of the length of the spring or variation of the spring stiffness.

**Figure 4:**
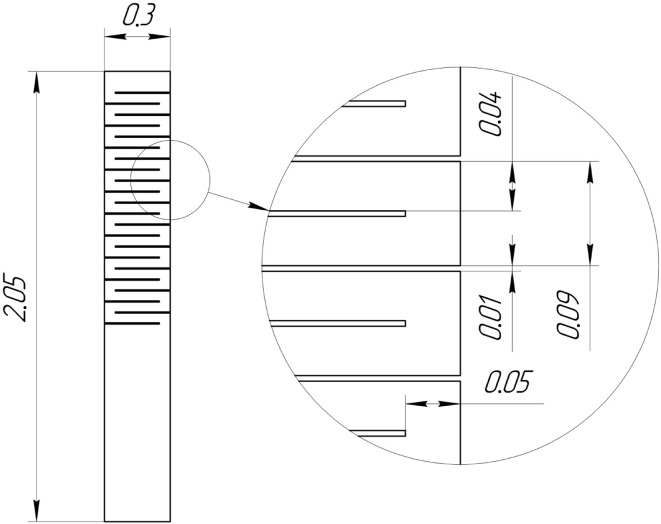
Scheme of the 15 µm-thick CVD spring. The inset is an enlarged view showing perforation dimensions. All dimensions are given in millimetres.

Increasing the length of the spring was found to be insufficient in our experiments to reach very small forces to minimize mounting strain in diamond crystal plates with ∼100 µm thickness. An additional spring force reduction was achieved by changing the stiffness of the CVD springs. The best solution to substantially reduce the stiffness of the springs was to introduce periodic strain-relief cuts (perforation) in the spring, as shown in Fig. 4 ▶. Finite element analysis was performed to evaluate the force exerted by either spring on the diamond plate (see supplementary information^1^). The force of the nonperforated spring was ∼
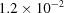
 N and that of the perforated spring was ∼
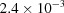
  N (*i.e.* reduced by a factor of 5).

All diamond components were manufactured at TISNCM using HPHT and CVD diamond synthesis methods and a controlled precision laser cutting of diamond materials. Polycrystalline CVD diamond films were deposited onto 640 µm-thick Si single-crystal substrates. Prior to deposition the Si substrates were ultrasonically treated in a diamond powder suspension in ethanol. The diamond deposition process was carried out in a bell-jar-type microwave plasma-assisted chemical vapor deposition reactor. The temperature of the Si substrates was maintained at 1133–1143 K. The methane concentration was 6% in a total flow of 500 s.c.c.m. (372 µmol s^−1^) The microwave power was in the range 3–3.2 kW at a total pressure of about 200 mbar (20 kPa) during deposition of thick films for the production of the 500 µm-thick CVD diamond substrates, and 130 mbar (13 kPa) during deposition of thin flexible films for the production of the 15 µm-thick CVD springs. After deposition the diamond films were separated from the Si substrates by dissolution of the substrates in a 44 wt% solution of KOH in water at 323 K.

**Figure 5:**
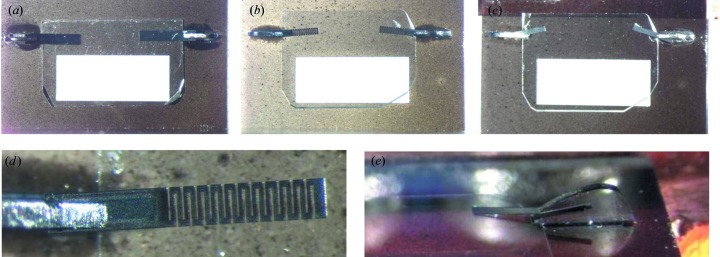
All-diamond assemblies (top view) with high-quality diamond crystal plates [type IIa, HPHT, (111) surface orientation] mounted on a CVD diamond substrate: (*a*) assembly with a 300 µm-thick diamond crystal plate mounted using nonperforated CVD diamond springs; (*b*) assembly with a 100 µm-thick diamond crystal plate mounted using perforated CVD diamond springs; (*c*) assembly with the same 100 µm-thick diamond crystal plate mounted using nonperforated CVD diamond springs. The bright rectangles in (*a*)–(*c*) are the 5 × 2 mm windows in the CVD substrates for passage of X-rays transmitted through the diamond plates. Selected microscope images: (*d*) an enlarged view of the perforated CVD diamond spring, and (*e*) a side perspective view of a diamond restrainer. A CVD spring that acts on the HPHT crystal is inserted into a groove in the restrainer.

The devices were assembled and cleaned at the Advanced Photon Source. Microscope images of the all-diamond assemblies are shown in Fig. 5 ▶. Fig. 5 ▶(*a*) shows an assembly with a 300 µm-thick diamond crystal plate mounted using nonperforated CVD diamond springs. Fig. 5 ▶(*b*) shows an assembly with a 100 µm-thick diamond crystal plate mounted using perforated CVD diamond springs. Fig. 5 ▶(*c*) shows an assembly with the same 100 µm-thick diamond crystal plate mounted using nonperforated CVD diamond springs.

Fig. 5 ▶(*d*) shows an enlarged view of the perforated spring acting on the 100 µm-thick diamond crystal plate, and Fig. 5 ▶(*e*) shows an enlarged side perspective view of a restrainer with an inserted CVD spring.

## 4. Characterization of mounting-induced strain   

### 4.1. Experimental   

**Figure 6:**
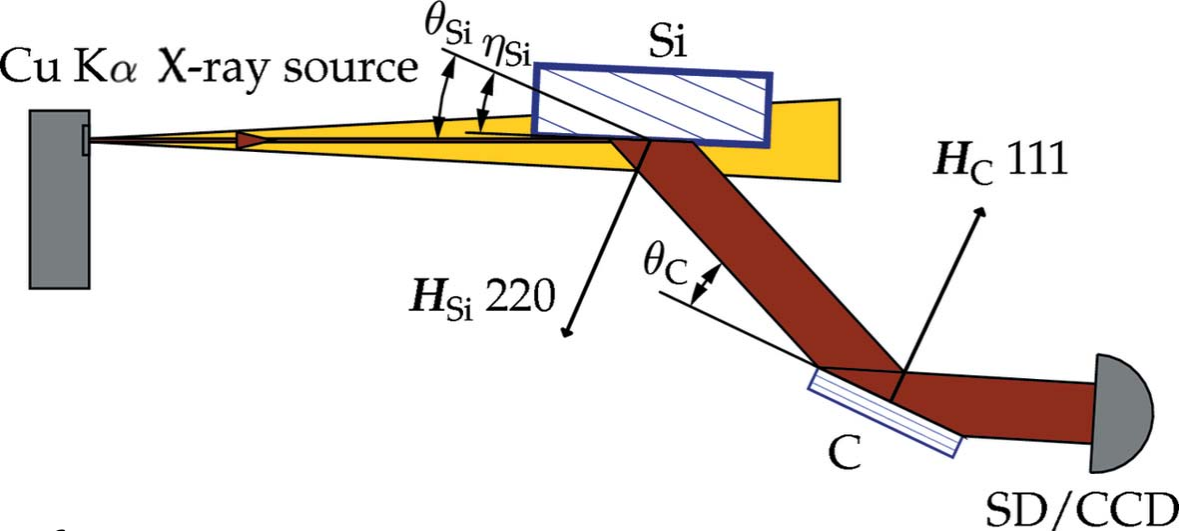
Double-crystal topography setup for mapping the rocking curve of the 111 diamond reflection (see text for details).

Rocking curve measurements and rocking curve imaging  (Lübbert *et al.*, 2000 ▶) were the main diagnostic methods for evaluating the mounting-induced crystal strain in the all-diamond assemblies. Double-crystal topography using a Cu *K*α rotating anode X-ray source was performed to map the rocking curve of diamond crystal plates mounted in the holder assemblies. An Si(220) first crystal with an asymmetry angle 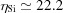
° was used to collimate an X-ray beam incident on the diamond crystal plate (C) mounted in the all-diamond assembly (Fig. 6 ▶). The diamond crystal was set for the 111 reflection. The Bragg angle of the diamond crystal was 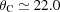
°, which was close to the Bragg angle of the collimator crystal 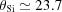
°.

In order to align the diamond crystal for the 111 reflection and to obtain the rocking curve of the entire crystal (total rocking curve), the intensity reflected from a uniformly illuminated diamond crystal was measured using a scintillation detector (SD). A CCD camera (with a resolution of 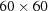
 µm) replacing the SD was used to obtain a series of X-ray diffraction images at different angular positions of the diamond crystal through the rocking curve of the 111 reflection. The image data were sorted such that a local rocking curve was obtained for every pixel, thus making it possible to map the rocking curve parameters over the entire crystal (*i.e.* rocking curve imaging). The angular resolution in the rocking curve scan was ∼4 µrad, which is small compared to the expected FWHM values.

At the Cu *K* 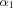 photon energy (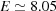
 keV) the intrinsic angular width of the diamond 111 rocking curve (*i.e.* Darwin width) is about 23 µrad for a monochromatic wave with σ polarization and ∼17 µrad for a monochromatic wave with π polarization. The FWHM of the ideal rocking curve of the diamond crystal in the utilized double-crystal configuration (assuming ideal collimation of the X-ray beam after the Si 220 reflection) can be estimated as follows (see *e.g.* Zachariasen, 1945 ▶):
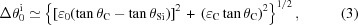
where 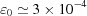 is the relative spectral width of the incident radiation (Cu *K* 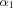) and 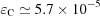 is the relative intrinsic bandwidth of the diamond 111 reflection (for *e.g.* σ polarization). The resulting estimate 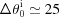
 µrad was verified using the dynamical theory of X-ray diffraction assuming a uniform angular distribution of the radiation incident on the first crystal (over an angular range exceeding the angular acceptance region of the crystal) and a photon energy distribution of the Cu *K*α characteristic lines (Härtwig *et al.*, 1993 ▶). The FWHM values were obtained by averaging the results of calculations for the two polarizations for the 100 µm-thick diamond crystal (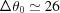
 µrad) and for the 300 µm-thick diamond crystal (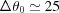
 µrad).

### 4.2. Analysis of total and local rocking curves   

**Figure 7:**
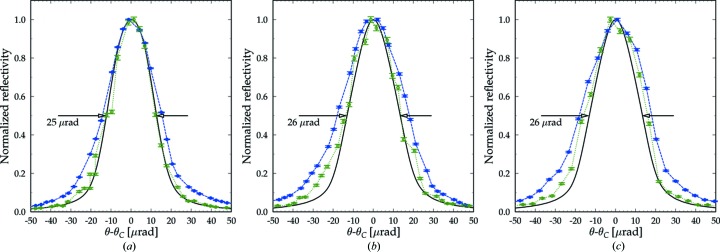
Rocking curves of diamond crystal plates in the all-diamond assemblies: total (filled blue circles, dashed blue line), local (open green circles, dotted green line) and theoretical (solid black line) for (*a*) a 300 µm-thick plate mounted using nonperforated CVD diamond springs; (*b*) a 100 µm-thick plate mounted using perforated CVD diamond springs; and (*c*) a 100 µm-thick plate mounted using nonperforated CVD diamond springs.

Fig. 7 ▶ shows measured total rocking curves (blue circles and lines), the theoretical rocking curve (solid black line) and local rocking curves (green circles and lines) from a selected pixel at the center of the working region on the diamond (111) crystal plate. The rocking curves are normalized to facilitate analysis of the curve widths. The experimental curves were also compared on the same intensity scale. A reasonable agreement in reflectivity was found for the thick and thin crystal plates, as expected in theory (see supporting information).

Fig. 7 ▶(*a*) shows rocking curves for the 300 µm-thick crystal mounted using nonperforated CVD diamond springs (as shown in Fig. 5 ▶ *a*). The FWHM of the local rocking curve matches that of the theoretical curve (25 µrad), while the total curve exhibits an additional broadening. This observation suggests that the broadening of the total rocking curve is caused by the presence of defects in the crystal (*i.e.* intrinsic strain). The defects are located outside the working region of the crystal, as will be shown below.

Fig. 7 ▶(*b*) similarly shows rocking curves for the 100 µm-thick crystal mounted using perforated CVD diamond springs (as shown in Fig. 5 ▶ *b*). In this case, both the total rocking curve and the local rocking curve exhibit an additional broadening compared to the FWHM of the theoretical rocking curve for the 100 µm-thick crystal (26 µrad).

Fig. 7 ▶(*c*) shows rocking curves for the 100 µm-thick crystal mounted using nonperforated CVD diamond springs. The broadening of the local rocking curve becomes somewhat larger, which suggests that the use of perforated springs is preferable for minimization of strain. However, this observation needs an additional verification, since there could be a variation in the local rocking curve from one pixel to another.

The values of FWHM of the measured total and local rocking curves and those of the theoretical rocking curves are given in Table 1 ▶ for each case. The FWHM of the total rocking curve (36 µrad) is about the same for (*b*) and (*c*), which indicates that the broadening is dominated by crystal defects and that this parameter is not very sensitive to mounting strain. The broadening is smaller than the intrinsic angular width (Darwin width) of the reflection (∼23 µrad for a monochromatic wave with σ polarization at the Cu *K*α photon energy). For certain applications of diamond in X-ray optics this validates the quality of the entire crystal and the mounting method using either springs. However, in our case the desired level of broadening should be less than or comparable to the angular divergence of the XFEL beam (1–2 µrad r.m.s.) to minimize disturbance of the radiation wavefront. Therefore, a more detailed characterization of the working crystal region is required.

### 4.3. Analysis of the rocking curve topographs   

The comparative analysis of the widths of the total and local rocking curves does not show a complete picture since it does not take into account the distribution of the rocking curve parameters over the crystal plate. A more sensitive indicator of the crystal strain is a local change in the lattice parameter or a tilt of the Bragg plane, which lead to a shift in the peak position of the local rocking curve. In our diffraction imaging analysis this shift is represented by the variation of the center of mass (COM) of the local rocking curve.

**Figure 8:**
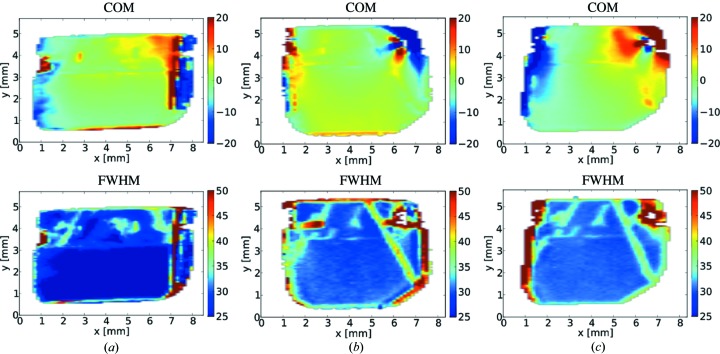
Double-crystal topographs showing maps of the rocking curve COM and the FWHM for (*a*) the 300 µm-thick crystal mounted using nonperforated CVD diamond springs; (*b*) the 100 µm-thick crystal mounted using perforated CVD diamond springs; and (*c*) the 100 µm-thick crystal mounted using nonperforated CVD diamond springs. The units on the color bars are given in µrad.

Fig. 8 ▶ shows maps (topographs) of the rocking curve COM and FWHM for the 300 µm-thick plate mounted using the nonperforated springs (*a*), the 100 µm-thick plate mounted using the perforated springs (*b*), and the 100 µm-thick plate mounted using the nonperforated springs (*c*). These maps resemble the white-beam topographs given above. In particular, the higher crystal quality in the working region and the diagonal lines (note the higher contrast in the FWHM map) representing growth sector boundaries are clearly observed. The COM topographs indicate that a substantial strain is present in the upper part of the crystals. In the COM topographs for (*a*) and (*b*) the contrast is predominantly due to the intrinsic strain (presence of defects). In case (*c*) the COM gradient is stronger, which indicates the presence of mounting-induced strain.

**Figure 9:**
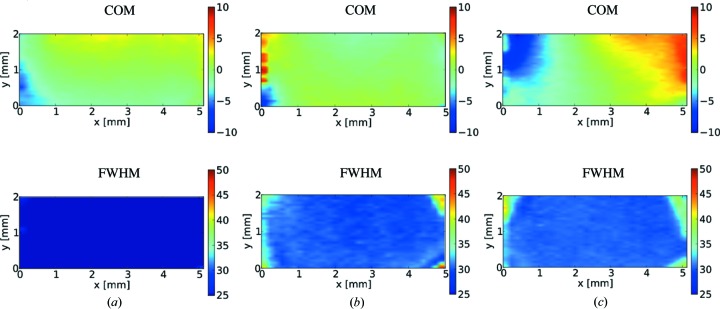
Double-crystal topographs (COM and FWHM) of the working crystal regions (∼5 × 2 mm) for (*a*) the 300 µm-thick crystal mounted using nonperforated CVD diamond springs; (*b*) the 100 µm-thick crystal mounted using perforated CVD diamond springs; and (*c*) the 100 µm-thick crystal mounted using nonperforated CVD diamond springs. The units on the color bars are given in µrad.

The topographs of the working region are shown separately in Fig. 9 ▶. The distribution of the FWHM is fairly uniform across the working region in each case. The COM topographs for (*a*) and (*b*) are again quite uniform, while in case (*c*) a distinct gradient is observed. The topographs of the working region illustrate that for the 300 µm-thick plate mounting it is acceptable to use nonperforated springs (Fig. 9 ▶ *a*). However, for the 100 µm-thick plate mounting use of the nonperforated springs results in a higher level of strain (Fig. 9 ▶ *c*) compared to the case of perforated springs (Fig. 9 ▶ *b*).

A simple statistical analysis of the rocking curve parameters was performed across the working region in each case. The values of the standard deviation of the COM, the standard deviation of the FWHM and the average value for the FWHM are given in Table 2 ▶. The largest variation of the local rocking curve peak position (COM) was for the 100 µm-thick plate mounted using nonperforated CVD springs (3.5 µrad). This value is still small compared to the average FWHM (31 µrad), which ensures a reasonable performance of the crystal in Bragg diffraction. The use of the perforated diamond CVD springs yields further minimization of the mounting-induced strain, as reflected by the reduced standard deviation of the COM of the local rocking curve (1.5 µrad).

## 5. Performance of the beam-splitting monochromator at the LCLS   

The all-diamond assemblies were installed in the double-crystal monochromator at the XPP instrument of the LCLS. The design of the monochromator permits a change from diamond (111) to Si(111) crystals. A full description of the performance of the diamond (111) beam-multiplexing monochromator at the LCLS is reported in a separate publication (Zhu *et al.*, 2014 ▶). Here, the main performance parameters are briefly summarized to conclude our study and to validate the design of the all-diamond assemblies.

The averaged spectrum of the transmitted branch was evaluated using a bent-crystal single-shot spectrometer (Zhu *et al.*, 2012 ▶). The monochromatic branch was characterized at 7.9 keV using an Si(440) crystal as an analyzer and by the rocking curve of the second (300 µm-thick) diamond crystal. The throughput of the monochromator in the pink and the monochromatic branches was measured and was found to be close to the expected theoretical values. The throughput of the monochromatic branch was normalized to the throughput of the same double-crystal monochromator using Si(111) crystals at the same photon energy.

Detailed characterization of the radiation wavefront remains challenging because of shot-to-shot pointing fluctuations of the FEL beam and because of beam profile structure originating at the front-end hard X-ray mirrors at the LCLS. No significant distortion in the beam intensity profiles was observed in either the pink or the monochromatic branch of the beam-multiplexing monochromator.

The measured values are summarized in Table 3 ▶. In addition, the temperature distribution in the all-diamond assembly of the first crystal was evaluated under the operating conditions using an infrared camera (see supporting information). It was found that the temperature variation in the all-diamond assembly does not exceed ∼3 K, while the temperature distribution in the crystal remains fairly uniform.

## 6. Conclusions   

In summary, all-diamond X-ray optical assemblies holding type IIa diamond (111) crystal plates were fabricated for the beam-multiplexing diamond monochromator at the LCLS. It is demonstrated how requirements on crystal quality and crystal mounting lead to an advanced application of diamond materials in XFEL optics. Two crystal plates of 100 and 300 µm thicknesses with a low concentration of defects in a region of 5 × 2 mm were selected using white-beam X-ray topography. A dedicated crystal mounting method was developed for minimization of mounting strain. The mounting strain was evaluated using double-crystal X-ray topography in the rocking curve imaging mode. The variation of the rocking curve peak position over the working region of the crystal plates was found to be substantially smaller than the angular width of the curve and comparable to the angular divergence of the XFEL beam. With these assemblies installed in the double-crystal monochromator at the LCLS, the capability of splitting the XFEL beam into a pink and a monochromatic branch was demonstrated (Zhu *et al.*, 2014 ▶). It was found that the measured bandwidth and throughput of the monochromator closely match the theoretical values, and the resulting beam profiles are only minimally disturbed.

## Supplementary Material

Supporting information file. DOI: 10.1107/S1600576714013028/nb5110sup1.pdf

## Figures and Tables

**Table 1:** Total (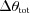), local (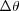) and theoretical (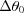) rocking curve FWHMs for assemblies (*a*), (*b*) and (*c*) (Fig. 5 ▶) with different diamond (111) crystal plate thicknesses (*t*) and CVD springs

Assembly	*t* (µm)	CVD springs	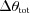 (µrad)	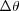 (µrad)	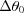 (µrad)
(*a*)	300	Nonperforated	30	25	25
(*b*)	100	Perforated	36	28	26
(*c*)	100	Nonperforated	36	32	26

**Table 2:** Rocking curve statistical parameters across the working region for assemblies (*a*) (*b*) and (*c*) (Fig. 5 ▶) 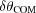
: standard deviation of the local rocking curve COM; 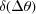: standard deviation of the local rocking curve FWHM; 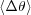: average value of the local rocking curve FWHM.

Assembly	*t* (µm)	CVD springs	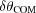 (µrad)	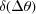 (µrad)	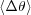 (µrad)
(*a*)	300	Nonperforated	1.4	0.4	25
(*b*)	100	Perforated	1.4	1.5	31
(*c*)	100	Nonperforated	3.5	1.5	31

**Table 3:** Performance parameters of the double-crystal beam-multiplexing monochromator at the LCLS 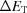
: the width of a spectral notch in the transmitted averaged spectrum at 7.9 keV; *T*: throughput of the transmitted branch at 7.1 keV; 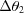: FWHM of the rocking curve of the second diamond crystal at 7.9 keV; 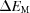: FWHM of the rocking curve of the Si(440) analyzer at 7.9 keV (in units of photon energy); 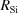: throughput of the monochromatic branch normalized by the throughput of the Si(111) double-crystal monochromator at 7.9 keV.

Parameter	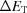 (eV)	*T*	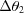 (µrad)	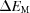 (eV)	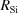
Experiment	0.5	0.57	27.5	0.35	0.47
Theory	0.43	0.59	24.4	0.34	0.47

## Footnotes

1 Supporting information for this paper is available from the IUCr electronic archives (Reference: NB5110). See also http://python-dtxrd.readthedocs.org.
