# Supplementary material for: All-diamond optical assemblies for a beam-multiplexing X-ray monochromator at the Linac Coherent Light Source
Source: J Appl Crystallogr. 2014 Aug 1;47(Pt 4):1329–36. doi: 10.1107/S1600576714013028 (PMC4119950; doi:10.1107/S1600576714013028)
Supplement: Supplementary file 1 [file j-47-01329-sup1.pdf]

Supplementary information

I. FINITE-ELEMENT ANALYSIS OF THE PERFORATED CVD SPRING

Finite-element simulations of strain distribution in the non-perforated and the perforated springs were performed using ANSYS® program. Using finite-element simulation a linear relation between the spring deformation and the exerted force was found. It was established that for the maximum deflection of the spring in the all-diamond assembly ($\simeq 350$ μm) the force exerted on the diamond plate is about 1.2×10^{-2} N for the non-perforated (plain) string and 2.36×10^{-3} N for the perforated spring. The calculated profiles of the loaded springs are shown in Fig. 1.

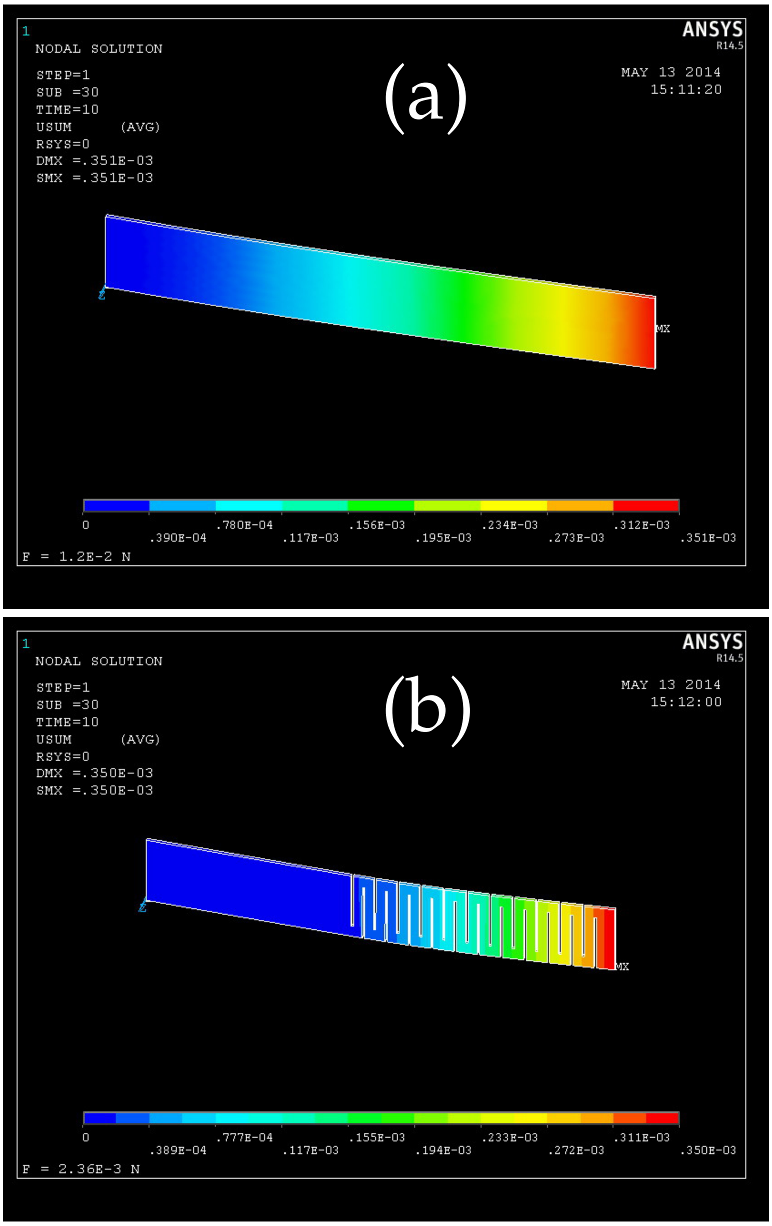

FIG. 1: Calculated deflection profiles of the loaded CVD springs: non-perforated (a) and perforated (b). The colorbars are in units of m.

II. COMPARISON OF REFLECTIVITIES OF THE DIAMOND CRYSTALS

For the purpose of complete crystal characterization comparison of non-normalized reflectivities provides information complementary to that given by the widths and peak positions of the curves. Such comparison is presented below.

A. Comparison of the theoretical curves

At the Cu K_α photon energy ($\approx$ 8 keV) of the double-crystal topography setup used in this work the extinction length for the diamond 111 reflection is about 1 μm (i.e., the effective penetration length of x-rays into the crystal depth at the center of the Bragg reflection region). Therefore, both the 300-μm-thick and the 100-μm-thick diamond crystals can be treated under the thick-crystal approximation where the reflection intensity within the Bragg reflection region given by the Darwin solution [1] (often referred to as Darwin table) is essentially the same. Minor differences in theory can arise due to averaging of thickness oscillations on the tails of the intrinsic reflectivity curve of the diamond 111 reflection. The intrinsic curves are shown in Fig. 2(a) as reflectivity for an incident monochromatic wave with σ-polarization. The period of the oscillations is greater for the 100-μm-thick perfect diamond crystal which results in a minor broadening of the rocking curve if convoluted with a smooth function (e.g., a Gaussian curve).

Figure 2(b) shows theoretical rocking curves for a 300-μm-thick perfect diamond crystal (blue line) and a 100-μm-thick perfect diamond crystal (green line). Calculations were performed using DTXRD codes [2] in the Si 220 - C 111 double-crystal geometry by propagating an ensemble of monochromatic waves through the analytical expressions of the dynamical theory of x-ray diffraction (2-beam approximation) and averaging results for the two polarizations. A minor difference between the curves is observed (a slight broadening and a minor reduction in intensity for the green curve).

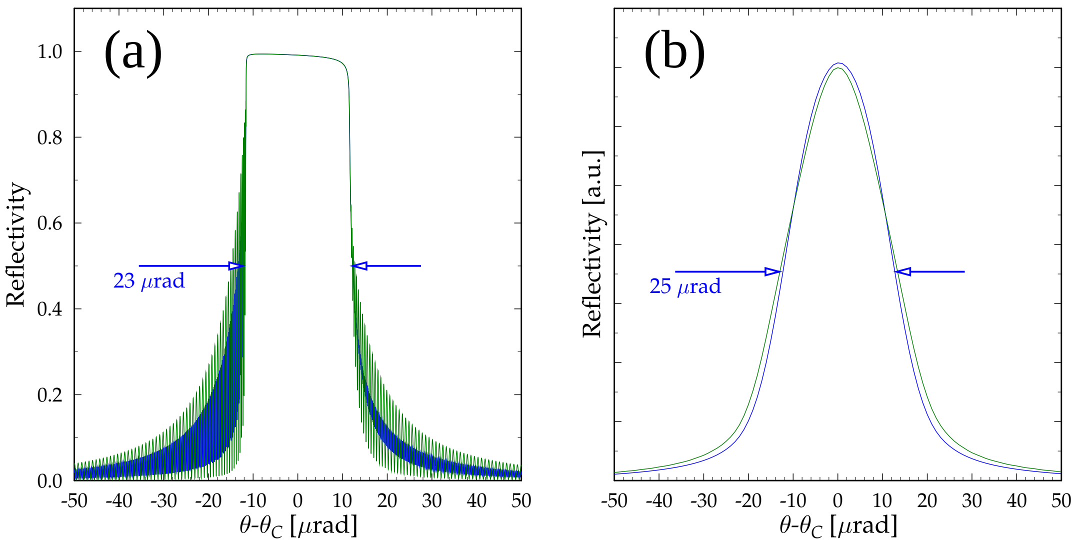

FIG. 2: Theoretical rocking curves for a 300-μm-thick diamond crystal (blue line) and a 100-μm-thick diamond crystal (green line): (a) intrinsic reflectivity curves for a monochromatic incident wave with σ-polarization; (b) calculations performed in the Si 220 - C 111 double-crystal geometry using DTXRD codes (see text for details).

B. Comparison of the experimental curves

Figure 3 shows the measured total rocking curves in raw units (counts) for the diamond plates under the different mounting conditions. We note that our experimental setup did not have a monitor of intensity incident on the diamond crystals. Thus, comparison of the reflectivities on the same scale has to rely on the stability of the flux generated by the rotating anode source . The measured reflectivities are nearly the same (as expected in theory for perfect crystals) except the one for the 100-μm-thick plate mounted using non-perforated CVD springs. In this case the likely conclusion is that the mounting-induced strain results in the reduction of the total reflectivity.

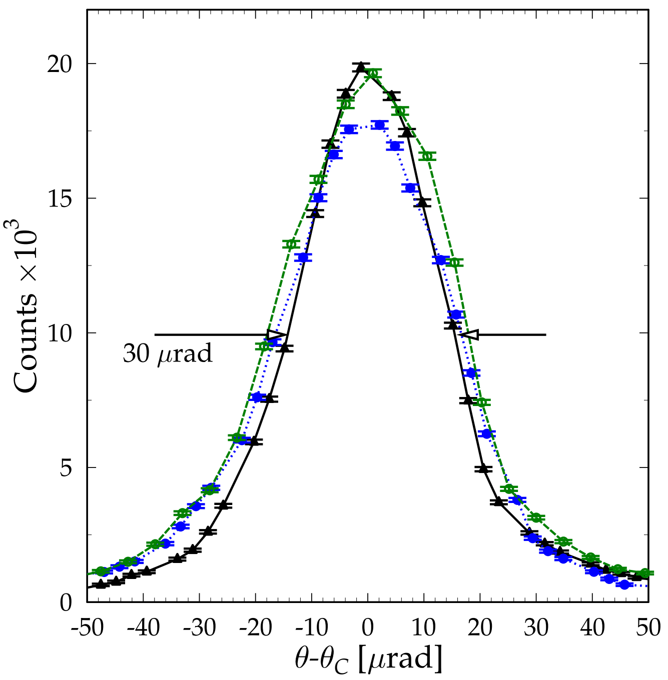

FIG. 3: Measured total rocking curves for the 300-μm-thick plate mounted using non-perforated CVD springs (black triangles, solid black line), the 100-μm-thick plate mounted using non-perforated CVD springs (filled circles, dotted blue line) and the the 100-μm-thick plate mounted using perforated CVD springs (open circles, dashed green line).

III. TEMPERATURE DISTRIBUTION IN THE ALL-DIAMOND ASSEMBLY UNDER THE HEAT LOAD OF XFEL RADIATION

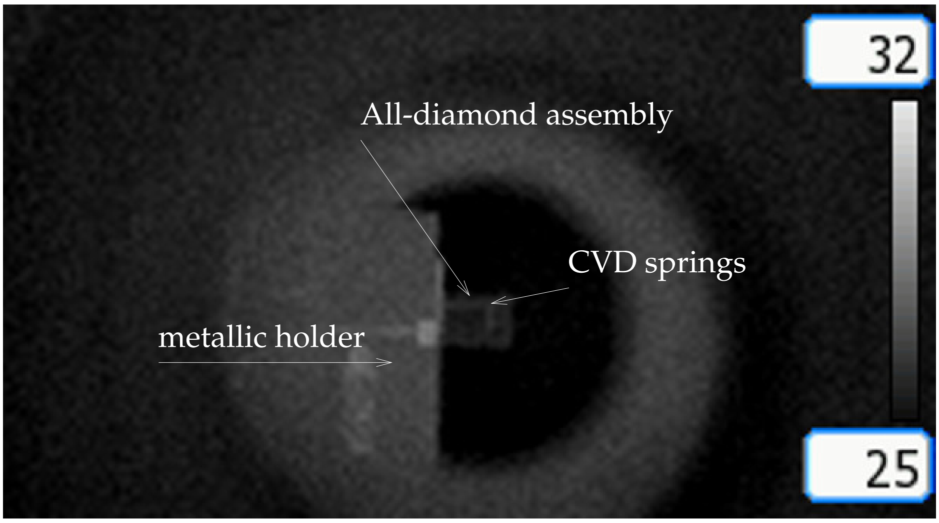

FIG. 4: Temperature distribution in the all-diamond assembly captured using an infrared camera. The grayscale bar is in units of C°.

Heat load is not a primary concern for diamond optics illuminated with the x-ray beam generated by the LCLS where the average incident power density is ≈ 2 W/mm^2 (Typical SASE x-ray pulse energy is 2-3 mJ at the repetition rate of 120 Hz). In contrast, the power density incident on high-heat-load diamond optics at third-generation synchrotron sources is about 50-100 W/mm^2.

Temperature distribution in the all-diamond assembly of the first crystal was monitored using IR camera during operation of the beam-multiplexing monochromator at the LCLS. A snapshot of the distribution is shown in Fig. 4. The all-diamond assembly is located in the central part of the image. The image has poor spatial resolution, however, individual features such as location of diamond springs are noticeable due to slightly elevated temperature. The limits on the grayscale bar (in units of C°) suggest that the maximum temperature variation in the all-diamond assembly is ≈ 3 C° while the temperature distribution across the working diamond (111) crystal remains fairly uniform.

[1] W. H. Zachariasen, *Theory of X-Ray Diffraction in Crystals* (John Wiley & Sons, Inc., New York, 1945), reprinted by Dover Publications, New York, 1967.
[2] S. Stoupin (2014), (to be published, http://python-dtxrd.readthedocs.org).
